# Surgical treatment of secondary cicatricial alopecia of scalp and eyebrow

**DOI:** 10.4103/0970-0358.53014

**Published:** 2009

**Authors:** Ahmed Sabry Hassan

**Affiliations:** Department of General Surgery, Faculty of Medicine, Reconstructive and Plastic Surgery Division, Al-Azhar University Hospital, Egypt

**Keywords:** Alopecia, Secondary cicatricial, Scalp defects

## Abstract

Secondary cicatricial alopecia occurs as a result of destruction of hair follicles by scar tissue formed in the scalp and eyebrows. It is a permanent condition and regrowth of hairs in the area is not expected. The purpose of the study was to select the appropriate method for treating cicatricial alopecia. 24 patients were admitted to our hospital during the period from June 2006 to July 2007. They were suffering from acquired cicatricial alopecia affecting the scalp and the eyebrow. Their ages ranged from 6-48 years with mean age 26-25 years. They were treated surgically by total excision of the lesions with direct closure of the defect in ten cases, excision of alopecia with advancement flaps with the aid of scalp expanders in seven cases, scalp reduction through serial excision of alopecia in three cases and excision of alopecia and reconstruction of the defect by strip composite hair-bearing scalp grafts in four cases. Our results suggest there are three key factors that decide the surgical methods for treating alopecia: size, location and shape. We also discuss and evaluate the various techniques of reconstruction. Good results were obtained in 18 patients.

## INTRODUCTION

The scalp is probably the second most visible part of the human anatomy second only to the face. Aesthetic considerations are extremely important in devising any plan for the restitution of the scalp.[1] The eyebrows are a very noticeable structure and make an essential contribution to facial beauty.[2] Areas of alopecia may result from traumatic, thermal, radiation, neoplastic or infective process.[3] The surgical replacement of hair has progressively become an important part of the practice of plastic surgery. [4] Alopecia following extensive scarring of the scalp can be treated by transferring hair bearing parieto-occipital flaps if convenient or adjacent hair-bearing scalp after tissue expansion.[5] Awaiting stem cell research in this vital field of aesthetic surgery there is no known method to create new hair, and all current techniques for hair restoration involve redistributing the patient's existing hair.[6] Many techniques were used for treating alopecia surgically such as scalp reduction, hair grafting, the use of local flaps and the use of tissue expanders.[7]

## MATERIAL AND METHODS

24 patients were admitted to our hospital suffering from acquired cicatricial alopecia affecting the scalp and the eyebrow, during the period June 2006 to July 2007. Their ages ranged from 6-48 years with mean age 26.25 years. There were 14 males and 10 females and the interval from the initial injury to the time of reconstruction was between one and seven years. The lesions involved more than 30% of the scalp or the entire eyebrow length. The size of alopecia ranged from 2.5 to 25 cm^2^ with mean size11.35 cm^2^.

The causes of alopecia were postburn injury (15 cases), to mechanical trauma (5 cases) and pyogenic infection (4 cases).

Management of patients included evaluation of their medical conditions, defect analysis and assessment of surgical options. Skin biopsy was done to confirm the scarring process.

The patients were classified according to the method of covering the defect after excision of the alopecia into:-

**Figure 1:**
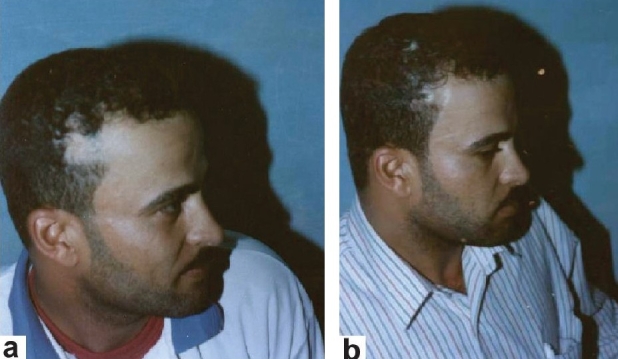
A 38 year-old man with posttraumatic alopecia of right temporal region (a) preoperative view (b) postoperative view excision of alopecia with direct closure of the defect

**Group 1:** Total excision of the lesions with direct closure of the defect (six cases) [Figure 1].

**Group 2:** Excision of alopecia and closure of the defect by rotation flap (three cases).

**Figure 2:**
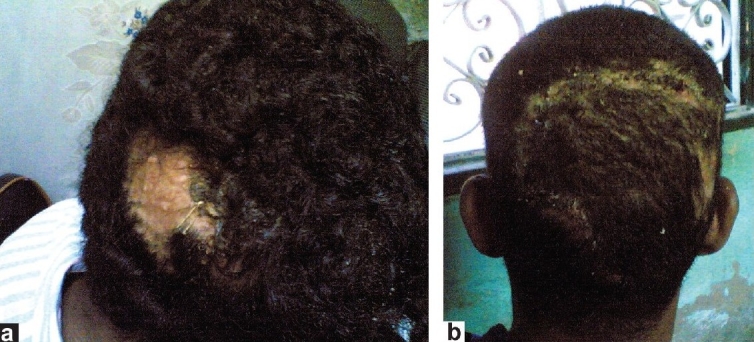
A 25-year-old man with postburn cicatricial alopecia of the occipital region (a) preoperative posterior view showing expansion of nearby skin (b) postoperative two-months posterior view showing viability of the flap after expander removal

**Group 3:** Excision of alopecia and closure of the defect with advancement flaps with the aid of scalp expanders (five cases) [Figure 2].

**Group 4:** Scalp reduction through serial excision of alopecia (three cases).

**Figure 3:**
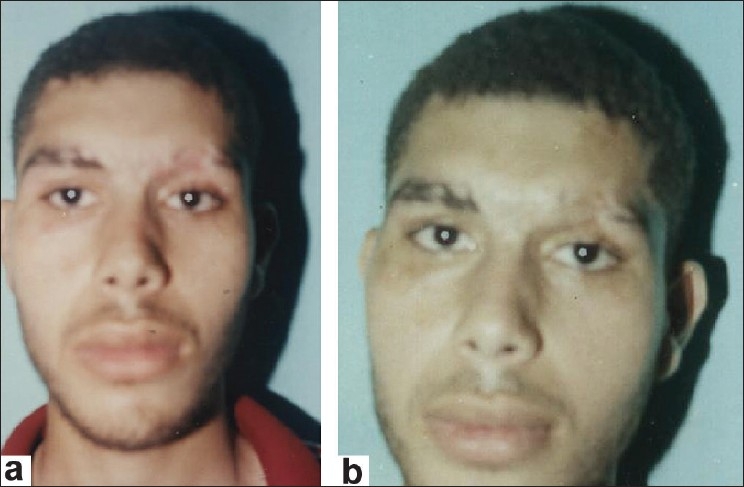
A 23 year-old man with postburn cicatricial alopecia of left eyebrow (a) A- preoperative view; (b) A- postoperative view

**Group 5:** Excision of alopecia and reconstruction using composite hair-bearing scalp strip graf ts (seven cases) [Figure 3].

**Technique:** All operations were done under general anesthesia. After sterilization of the area with 10% povidone iodine, infiltration with a local anesthetic (0.5% lidocaine with 1:200,000 epinephrine) was done to minimize the blood loss. The lesions were totally excised as in groups 1, 2 and 5 but in group 3, insertion of expander was done in the first sitting and once the desired expansion was achieved the alopecia was excised and the raw area was covered by a flap harvested from the expanded skin in the second stage. In group 4, serial excisions from the periphery of the lesion were done. Coverage of the defect thus was achieved by either direct approximation of two edges, or advancement flap with the aid of scalp expanders, or strip composite hair-bearing scalp grafts from the lower occipital and parietal areas of the scalp or scalp reduction through serial excision depending on the size of the defect and its location.

In reconstructing the eye brow defect by strip graft the hairs were oriented to grow upward and outward, as in a normal eyebrow.[8] The skin was closed with interrupted 4-0 nylon sutures. The dressings were changed for the first time at 3^rd^ postoperative day.

Criteria of evaluation was surgeon's satisfaction, patient's satisfaction, and the presence or absence of complications.

## RESULTS

Patients have been followed up for six months. The morning after the surgery, dressings were removed and the surgical site was cleansed gently with saline solution. Most patients healed and shed the scabs in 7-14 days. In group 5, there was an initial false growth of hair for approximately three to four weeks, subsequently those hairs shed and there was no growth of hair up to two to three months. Thereafter the hairs reappeared and significant growth of hair was noted at six month after surgery. In group 1 widening of suture line after total excision with direct closure was noted in one case and accepted by the patient.

Group1: Total excision of the lesions with direct closure of the defect (six cases). [Figure 1a and b]

Group 2: Excision of alopecia and closure of the defect by rotation flap (three cases). In this group necrosis of tip of the flap occurred in one case.

Group 3: Excision of alopecia and closure of the defect with advancement flaps with the aid of scalp expanders (five cases) [Figure 2a and 2b]. In this group exposure of expander, implant failure and infection occurred in three cases.

Group 4: Scalp reduction through serial excision of alopecia (three cases).

Group 5: Excision of alopecia then reconstruction of the defect by strip composite hair-bearing scalp grafts (seven cases) [Figure 3a and b]. In this group partial take of grafts were seen in three cases [Table 1]. Assessment of the results was good in 75% of patients [Table 1]. Results are also depicted in [Figures 1–3].

**Table 1:** Clinical series

*Sex*	*Age (years)*	*Site*	*Size cm^2^*	*Etiology*	*Procedure*	*Complication*	*Result*
Male	24	Occiput	25	Postburn	Expander	None	Good
Male	26	Occiput	15	Postburn	Rotation flap	None	Good
Male	48	Frontal	2.5	Trauma	Direct closure	None	Very good
Male	27	Rt temporal	3	Infection	Direct closure	None	Good
Male	16	Vertex	25	Postburn	Serial excision	None	Good
Female	39	Lt eyebrow	6	Postburn	Graft	Partial take of graft	Fair
Male	45	Occiput	8	Postburn	Rotation flap	None	Good
Male	17	Frontal	5	Postburn	Graft	None	Good
Male	48	Vertex	13	Trauma	Serial excision	None	Good
Male	7	Occiput	17	Infection	Rotation flap	Tip necrosis	Good
Female	35	Lt eyebrow	9	Postburn	Graft	None	Very good
Female	17	Lt temporal	16	Postburn	Expander	Exposure of expander	Fair
Female	37	Occiput	3	Infection	Direct closure	None	Very good
				Postburn	Serial excision	None	Good
Female	16	Lt eyebrow	7	Postburn	Graft	None	Good
Male	41	Rt frontotemporal	25	Trauma	Expander	Implant failure	Fair
Female	32	Occiput	3	Postburn	Direct closure	None	Good
Male	15	Rt frontal	5	Infection	Direct closure	None	Good
Female	18	Lt eyebrow	8	Postburn	Graft	Partial take of graft	Fair
Male	23	Rt eyebrow	7	Postburn	Graft	None	Good
Female	9	Rt Frontal	7	Trauma	Graft	None	Good
Female	11	Lt temporoparietal	16	Postburn	Expander	Infection and removale of expander	Fair
Female	30	Rt Parietal	25	Postburn	Expander	Exposure of expander	Fair
Male	24	Occiput	7	Trauma	Direct closure	None	Good

## DISCUSSION

Composite hair-bearing scalp grafting has proved to be an ingenious procedure for surgeon and patient alike because of its simplicity and versatility.[4] Dardour prefers flaps and reductions to grafts because of lesser number of operations and time required to obtain a good result. The results obtained by grafts are late and often unaesthetic.[9]

Scalping flaps are used for large defects not amenable to other forms of surgical treatment. However, they are often associated with long unsightly scars, unappealing skin grafts at donor sites and decreased sensation within the flaps.[10] The disadvantage of any scalp flap is that the donor defect is usually the same size as the flap.[11]

Scalp reduction is indicated for complete or partial elimination of alopecia on the vertex of the scalp.[4]

Tissue expansion is an important tool for providing donor skin that is an optimal match in terms of skin colour, texture and hair-bearing characteristics. This is achieved by recruiting local tissue, with primary closure at the donor site and minimal morbidity.[12] In large burn scar alopecia, due to lack of sufficient donor scalp, replacement with ‘like’ tissue is possible by expanded flaps. [11] Tissue expansion recruits both sensate and hair-bearing tissue into the recipient site when needed.[13]

In our study, the complication rate for tissue expansion was 60%. This agrees with Pandya *et al*, who observed that despite attempts to minimize complications and potential implant exposures the incidence of these complications remained high.[14]

Tissue expansion too has the disadvantage of being a two-stage procedure, multiple visits to the surgeon for repeated injections into the expander, infections, risk of skin necrosis and exposure of the expander. The cost of the expander and cost of commuting to the hospital several times should also be remembered while recommending tissue expansion. In addition, expanders cause an inelastic subdermal fibrous scar.[15]

Exposure of expanders may occur early in the course of expansion through a suture line or a preexisting scar, or occur late in the course of expansion.[16] Exposure of an expansion prothesis is most often related to: 1) inadequate dissection or an excessively large prothesis that abuts on the line of wound closure. This is the most frequent cause of early exposure, 2) excessively rapid and overzealous inflation 3) improper positioning of the implant. If local tissues are compromised by scarring but need to be expanded, a higher risk of exposure should be anticipated, and altered placement of expander or even alternate modalities of reconstructions should be seriously considered. This is particularly true in cases of irradiated and burned tissues.[17]

Leonard and Small, used the largest expander that could be accommodated.[12] However if an advancement flap is planned a rectangular expander should have a base at least as wide as the defect whereas a round expander needs to have an even larger base. Incisions should be along the sides of the expansion and outline a flap wider than the defect.[1,2]

Implant failure is relatively uncommon in experienced hands. We have seen leakage due to a puncture in the expander dome itself. Difficulty during expansion can occur if there is a problem in locating the expander valve. Tissue expansion can produce excellent results where indicated, but should be used as an adjunct to, and not a substitute for other reconstructive procedures.[18]

There are five factors to consider when planning a tissue expansion strategy: (1) the dimensions of tissue to be replaced, (2) proper expander selection, i.e. how big or which type, (3) incision type and placement for expander insertion, (4) the number of expanders to be used, and (5) the schedule for saline injections. Careful planning is necessary to expand tissue in the required direction and dimension to obtain excellent results. On the other hand, few complications are actually encountered during the expansion process itself and there is a lower rate of associated complications following reconstruction. Exposure of the tissue expander is one typical complication encountered using this method in our series. Pressure seems to be the cause of such expander exposure.[19] In our study the high complication rate of expander placement is related to our unfamiliarity with the technique in the early cases of this study.

## CONCLUSION

No better substitute for scalp tissue exists than scalp tissue itself. Tailoring the right procedure for the right patient is necessary for scalp coverage either with flap or skin expansion techniques.
